# Regulation of mTOR Signaling: Emerging Role of Cyclic Nucleotide-Dependent Protein Kinases and Implications for Cardiometabolic Disease

**DOI:** 10.3390/ijms241411497

**Published:** 2023-07-15

**Authors:** Fubiao Shi, Sheila Collins

**Affiliations:** 1 Division of Cardiovascular Medicine, Department of Medicine, Vanderbilt University Medical Center, Nashville, TN 37232, USA; 2 Department of Molecular Physiology and Biophysics, Vanderbilt University, Nashville, TN 37232, USA

**Keywords:** mTOR, cAMP, cGMP, PKA, PKG, *Raptor*, *Rictor*, obesity, fatty liver disease, cardiac hypertrophy

## Abstract

The mechanistic target of rapamycin (mTOR) kinase is a central regulator of cell growth and metabolism. It is the catalytic subunit of two distinct large protein complexes, mTOR complex 1 (mTORC1) and mTORC2. mTOR activity is subjected to tight regulation in response to external nutrition and growth factor stimulation. As an important mechanism of signaling transduction, the ‘second messenger’ cyclic nucleotides including cAMP and cGMP and their associated cyclic nucleotide-dependent kinases, including protein kinase A (PKA) and protein kinase G (PKG), play essential roles in mediating the intracellular action of a variety of hormones and neurotransmitters. They have also emerged as important regulators of mTOR signaling in various physiological and disease conditions. However, the mechanism by which cAMP and cGMP regulate mTOR activity is not completely understood. In this review, we will summarize the earlier work establishing the ability of cAMP to dampen mTORC1 activation in response to insulin and growth factors and then discuss our recent findings demonstrating the regulation of mTOR signaling by the PKA- and PKG-dependent signaling pathways. This signaling framework represents a new non-canonical regulation of mTOR activity that is independent of AKT and could be a novel mechanism underpinning the action of a variety of G protein-coupled receptors that are linked to the mTOR signaling network. We will further review the implications of these signaling events in the context of cardiometabolic disease, such as obesity, non-alcoholic fatty liver disease, and cardiac remodeling. The metabolic and cardiac phenotypes of mouse models with targeted deletion of *Raptor* and *Rictor*, the two essential components for mTORC1 and mTORC2, will be summarized and discussed.

## 1. Introduction

The mechanistic target of rapamycin (mTOR) kinase is a central regulator of cell growth and metabolism. It acts as a signaling node that integrates the external inputs and coordinates the major anabolic and catabolic pathways to maintain metabolic homeostasis [1]. The mTOR kinase is an evolutionarily conserved serine/threonine kinase and a member of the phosphoinositide kinase-related kinase (PIKK) family. It is the catalytic subunit of two distinct large protein complexes, mTOR complex 1 (mTORC1) and mTORC2. These two complexes are distinguished by their accessory proteins, sensitivity to rapamycin, and distinct substrates and functions [1].

**Figure 1:**
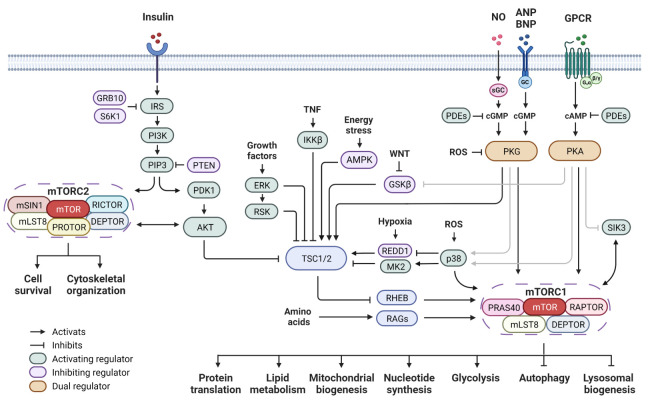
mTOR signaling network and cyclic nucleotide-dependent protein kinase pathway. Insulin acts through the PI3K pathway and leads to signaling cascades that further activate mTORC1 and mTORC2. Upstream regulators converge at the TSC complex to either activate or inhibit mTORC1 activity. The RAG GTPases are the key component of the amino acid sensing machinery. The second messenger signaling including cAMP and cGMP pathways regulates mTOR activity through both direct and indirect mechanisms. Abbreviations: mTOR: mechanistic target of rapamycin; RAPTOR: regulatory-associated protein of mTOR; RICTOR: rapamycin-insensitive companion of mTOR; DEPTOR: DEP domain-containing mTOR-interacting protein; mLST8: mammalian lethal with SEC13 protein 8; PRAS40: proline-rich AKT substrate of 40 kDa; mSIN1: mammalian stress-activated protein kinase-interaction protein 1; PROTOR: protein observed with RICTOR; TSC1/2: tuberous sclerosis complex 1/2; RHEB: Ras homolog mTORC1 binding; RAGs: Ras-related GTPases; IRS: insulin receptor substrate; PI3K: phosphoinositide 3-kinase; PIP3: phosphatidylinositol 3,4,5-triphosphate; PTEN: phosphatase and tensin homolog; PDK1: phosphoinositide-dependent protein kinase 1; GRB10: growth factor receptor bound protein 10; AKT: protein kinase B; ERK: extracellular signal-regulated kinase; RSK: ribosomal S6 kinase 1; TNF: tumor necrosis factor; IKKβ: inhibitor of nuclear factor kappa-B kinase subunit beta; AMPK: AMP-activated protein kinase; WNT: wingless and lnt-1; GSKβ: glycogen synthase kinase 3β; REDD1: regulated in development and DNA damage responses 1; p38 MAPK: p38 mitogen-activated protein kinases; MK2: MAPK activated protein kinase 2; ROS: reactive oxygen species; NO: nitric oxide; ANP: atrial natriuretic peptide; BNP: B-type natriuretic peptide; GPCR: G protein-coupled receptor; GC: guanylyl cyclase: sGC: soluble guanylyl cyclase; cAMP: cyclic adenosine monophosphate; cGMP: cyclic guanosine monophosphate; PKA: cAMP-dependent protein kinase; PKG: cGMP-dependent protein kinase; SIK3: salt-inducible kinase 3; PDEs: phosphodiesterases.

The regulatory-associated protein of mTOR (RAPTOR) and the rapamycin-insensitive companion of mTOR (RICTOR) are the defining components for mTORC1 and mTORC2, respectively (Figure 1). Rapamycin forms a complex with FKBP12 (FK506-binding protein of 12 kDa) and prevents the substrate’s entry into the kinase activity site and thus inhibits mTORC1 activity [2,3,4,5,6]. mTORC2 is relatively insensitive to acute rapamycin treatment. However, prolonged rapamycin treatment inhibits mTORC2 signaling due to the sequestering of the cellular mTOR pool into the rapamycin-bound complex [7,8]. It has been well established that mTORC1 plays essential roles to promote protein synthesis, lipid metabolism, mitochondrial biogenesis, nucleotide synthesis, and glycolysis, while suppressing autophagy and lysosomal biogenesis (reviewed in [1]). mTORC2 controls a spectrum of cellular functions including cell survival and cytoskeletal organization (reviewed in [1]).

mTOR activity is tightly regulated to sense the nutrients and growth factors to orchestrate cell growth and metabolic program. The integrated upstream signals that control mTORC1 activity mainly include two sets of small G proteins, RHEB (Ras homolog mTORC1 binding) and RAGs (Ras-related GTPases) (Figure 1). RHEB is located in the lysosomal surface and in its active GTP-bound state it is competent to activate mTORC1 kinase activity [9,10]. The RAGs GTPases are essential components of the amino acid sensing machinery. They promote the subcellular translocation of mTORC1 to lysosomes [11,12], where mTORC1 subsequently interacts with and becomes activated by RHEB. The tuberous sclerosis complex (TSC) is a complex of TSC1-TSC2 (hamartin–tuberin) and it acts as a GTPase activating protein to negatively regulate the lysosomal RHEB [13,14,15,16]. Upstream signaling kinases phosphorylate TSC, thus modifying TSC activity, and in turn stimulate (AKT, ERK, RSK-1, IKKβ) or inhibit (AMPK and GSK-3β) mTORC1 activity (Figure 1). mTORC2 is primarily activated by growth factors through the phosphoinositide 3-kinase (PI3K) pathway (Figure 1). Binding of phosphatidylinositol 3,4,5-triphosphate (PIP3) leads to mTORC2 activation [17,18,19]. The tumor suppressor PTEN (phosphatase and tensin homolog) inhibits mTORC2 activity by promoting the conversion of PIP3 to phosphatidylinositol 4,5-bisphosphate (PIP2) [20,21].

## 2. Cyclic Nucleotide-Dependent Kinase Regulation of mTOR Signaling

As briefly reviewed in Part 1, the mTOR signaling cascades are tightly regulated in response to external nutrition and growth factors to coordinate cell growth and metabolism. As an important mechanism of signaling transduction, the ‘second messenger’ cyclic nucleotides including cAMP and cGMP and their associated cyclic nucleotide-dependent kinases, including protein kinase A (PKA) and protein kinase G (PKG), have been shown to exert both positive and negative regulation of mTOR signaling in various physiological contexts and disease conditions. In the following part, we will summarize the earlier work establishing the ability of cAMP to dampen mTORC1 activation in response to insulin and growth factors and then discuss our recent findings demonstrating the regulation of mTOR signaling by the PKA- and PKG-dependent signaling pathways that represent a new non-canonical pathway independent of AKT, and we will review the implications of these signaling events in cardiometabolic disease, such as obesity, non-alcoholic fatty liver disease, and cardiac remodeling.

### 2.1. Regulation of mTOR Signaling by PKA

The seven transmembrane-spanning G protein-coupled receptors (GPCRs) are cell surface proteins that, upon agonist stimulation, can interact with heterotrimeric G proteins that in turn regulate various effector enzymes or ion channels (see [22] for review). GPCRs such as the β-adrenergic receptors (βAR), thyroid-stimulating hormone (TSH) receptor, and glucagon receptor interact with the Gs heterotrimer to stimulate adenylyl cyclase to convert ATP into the ‘second messenger’ cAMP. Other GPCRs can regulate inositol phosphate metabolism or inhibit cAMP production. Downstream events that respond to increases in cAMP include activating the cAMP-dependent protein kinases (PKA) as well as small G proteins such as exchange proteins directly activated by cAMP-1 (EPAC1) and EPAC2. The regulation of mTOR signaling by the cAMP/PKA pathway has been documented in many studies. However, as not appreciated by these previous studies, we have shown that cAMP can either inhibit or activate mTOR activity depending on the cell type, signaling context, and physiological conditions (summarized in Table 1).

Since mTOR was first determined to be involved in growth and responsive to growth factors, the first studies examining the effect of cAMP reported that it acts to antagonize the effect of insulin or growth factors on mTOR activity. These studies were mostly performed in cultured cell lines. Monfar and colleagues reported that reagents that increase cAMP and activate PKA inhibit the cytokine interleukin-2 (IL-2)-dependent activation of S6K1 in IL-2-responsive lymphoid cells [23]. Scott and Lawrence showed that increasing cAMP blocked the effect of insulin in 3T3-L1 adipocyte to activate mTOR, suggesting an antagonizing effect of cAMP on insulin action and mTOR activity [24]. Xie and colleagues further showed that elevation of cAMP in mouse embryonic fibroblasts (MEFs) and human embryonic kidney 293 (HEK293) cells inhibits insulin-stimulated mTORC1 activation via a PKA-dependent mechanism, and prolonged elevation in cAMP can also inhibit mTORC2 [25]. This cAMP-dependent inhibition of mTORC1/2 was proposed to be caused by the dissociation of mTORC1 and 2 and a reduction in mTOR catalytic activity [25]. Rocha and colleagues reported that, in Ret/PTC1-positive thyroid carcinoma cell TPC-1, cAMP markedly inhibits the Raf/ERK cascade, leading to mTOR pathway inhibition [26]. Kim and colleagues showed that, in several cell lines, the cAMP-dependent signaling pathway inhibits AKT activity by blocking the coupling between AKT and its upstream regulators, PDK, in the plasma membrane, leading to the inhibition of mTOR [27].

Studies have also been conducted in primary cell culture or physiologically relevant tissues. Mothe-Satney and colleagues showed that, in primary cultures of hepatocytes, glucagon increases the mTOR phosphorylation at Ser2448 as insulin or amino acids do, but glucagon inhibits the insulin- and amino acid-induced activation of S6K1 and phosphorylation of 4E-BP1 [28]. Another study by Kimball and colleagues showed that glucagon represses both basal and amino acid-induced phosphorylation of 4E-BP1 and S6K1 in perfused rat liver as an experimental model [29]. The repression is associated with the activation of PKA and enhanced phosphorylation of liver kinase B1 (LKB1) and the AMP-activated protein kinase (AMPK). However, this study also showed that glucagon simultaneously enhances phosphorylation of two S6K1 downstream effectors S6 and eukaryotic initiation factor 4B (eIF-4B) [29]. This effect was proposed to act through the activation of the ERK-RSK signaling pathway. In addition, Baum and colleagues showed that glucagon acts in a dominant manner to repress insulin-induced mTORC1 signaling in perfused rat liver [30]. In the presence of a combination of insulin and glucagon, AKT and TSC2 phosphorylation and PKA activity were all increased compared with controls. However, mTORC1 signaling was repressed compared with livers perfused with a medium containing insulin alone, and this effect was proposed to be associated with reduced assembly of the mTORC1-eIF3 complex. In another more recent study, Csukasi and colleagues reported that the parathyroid hormone (PTH) receptor, another GPCR coupled to cAMP and PKA activation, led to mTORC1 inhibition during skeletal development [31]. It is known that PKA can inhibit salt-inducible kinase 3 (SIK3) and the other two SIK family members [32,33]. It was proposed that PTH/PTHrP signaling can inhibit SIK3. In this case, these authors showed that inhibiting SIK3 prevents the proteasomal degradation of DEPTOR, a negative regulator of mTOR. In a recent study, our group identified that SIK3 is an mTORC1 substrate in brown adipocytes that interacts with RAPTOR and can be phosphorylated in a rapamycin-sensitive manner [34]. Thus, this SIK3-mTORC1 connection could be a new layer of the signaling network that might convey PKA action to mTOR signaling and deserves further investigation.

Several other studies have also linked cAMP with mTORC1 activation. For example, Kwon and colleagues reported that the GLP-1 (glucagon-like peptide-1) receptor agonist, Exenatide, dose-dependently enhanced phosphorylation of S6K1 in isolated rat islets through a glucose-dependent and cAMP-mediated mechanism [35]. They also showed that this process was rapamycin-sensitive and results in the alteration of intracellular calcium flux [35]. Brennesvik and colleagues reported that adrenalin potentiated an insulin-stimulated activation of AKT and S6K through a mechanism that depends on cAMP and Epac in skeletal muscle [36]. Moore and colleagues showed that cAMP increasing agents such as GLP-1 and forskolin lead to the S6 activation in the pancreatic beta-cell line MIN6 (mouse insulinoma cell line 6) and islets of Langerhans through a mechanism that depends on PKA [37]. Blancquaert and colleagues reported that TSH/cAMP activates mTORC1 in PC Cl3 rat thyroid cells, leading to phosphorylation of S6K1 and 4E-BP1. This effect was proposed to be caused in part by T246 phosphorylation of PRAS40, which was found as an in vitro substrate of PKA [38]. Palaniappan and Menon reported that human chorionic gonadotropin stimulates theca-interstitial cell proliferation and cell cycle regulatory proteins through a mechanism involved in the cAMP-dependent activation of AKT and the mTORC1 signaling pathway [39]. Kim and colleagues reported that cAMP phosphodiesterase 4D (PDE4D) is a binding partner of RHEB and acts as a cAMP-specific negative regulator of mTORC1 [40]. Under basal conditions, it is reported that PDE4D binds RHEB in a noncatalytic manner that does not require its cAMP-hydrolyzing activity and thereby inhibits the ability of RHEB to activate mTORC1. In this same report, the authors state that elevated cAMP levels disrupt the interaction of PDE4D with RHEB and increase the interaction between RHEB and mTOR. This enhanced RHEB–mTOR interaction induces the activation of mTORC1 and cap-dependent translation. This study proposed a new role for PDE4 as a molecular transducer for cAMP signaling to regulate mTOR activity.

In 2016, our group reported the novel observation that mTORC1 activation is required for the β-adrenergic stimulation of adipose tissue browning [41]. Pharmacological inhibition of mTORC1 or genetic deletion of its defining component RAPTOR in adipocyte impairs the brown and beige adipocyte expansion in response to β-adrenergic agonist treatment or cold temperature exposure [41]. Specifically, our study showed for the first time that PKA can directly phosphorylate in RAPTOR at Ser791 and mTOR at S1276, S1288, and S2112 [41], providing a novel signaling mechanism whereby PKA regulates mTOR signaling, and this is likely a major mechanism responsible for the previous reports of the cAMP activation of mTORC1. A cell-type-specific knock-in of a RAPTOR mutation that changes Ser791 into a phosphorylation-resistant Ala791 in mice has been made in our lab and will be reported on in the future. For these and other studies, the generation of an antibody that recognizes the phosphorylated S791 in RAPTOR will be extremely helpful.

### 2.2. Regulation of mTOR Signaling by PKG

Newer studies are demonstrating that, like PKA, the cGMP/PKG pathway can also control mTORC1 activity (Table 1). The cardiac natriuretic peptides (NPs) stimulate cGMP/PKG signaling through the action of guanyl cyclase-coupled NP receptor A (NPRA). Our group has shown that, like PKA, PKG phosphorylates Ser791 on RAPTOR [42], and related to this, the NP-stimulated adipose tissue browning [43] is impaired with the pharmacological inhibition of mTORC1 by rapamycin [42,43]. It will be interesting to determine whether the adipose tissue browning effect of NPs is impaired in mice with a phosphorylation-resistant Ser791Ala knock-in mutation. This will provide insight into the in vivo function of the S791 phosphorylation site in the context of the NP-regulated adipose tissue thermogenesis program.

PKG has also been reported to regulate mTOR signaling by phosphorylating other components of the mTOR network. Ranek and colleagues reported that PKG1 activates TSC2 by direct phosphorylation at Ser1365 and Ser1366 in cardiomyocytes, thereby inhibiting mTORC1 and promoting autophagy activity to counter adverse remodeling during cardiac stress [44]. In addition, studies have shown that PKG1α oxidation at cysteine-42 can be induced by oxidative stress [45]. Oeing and colleagues showed that the PKG1α cysteine-42 redox state controls mTORC1 activation in pathological cardiac hypertrophy [46]. The oxidation of PKG1α at C42 reduces its phosphorylation of TSC2, resulting in amplified pressure overload-stimulated mTORC1 activity and adverse cardiac remodeling [46]. As PKG1 is the major effector of both nitric oxide-generated cGMP and natriuretic peptide-stimulated cGMP, these studies provide mechanistic insights into the protective effects of these reagents against heart disease and oxidative stress.

### 2.3. Indirect Regulation of mTOR Signaling

As the major effector kinases of second messenger signaling, several reports have suggested that PKA and PKG can regulate mTOR activity through indirect mechanisms via several downstream signaling regulators, such as p38 mitogen-activated protein kinases (MAPKs) and glycogen synthase kinase 3 (GSK3) (Table 1).

p38 is a class of MAPKs that is responsive to stress stimuli and plays a central role in a diverse range of stress-induced signaling pathways [47,48]. Li and colleagues showed that p38 MAPK controls TSC2 Ser1210 phosphorylation through its downstream kinase MAPKAPK2 (MK2), which enhances TSC2 interaction with 14-3-3 proteins and in turn inhibits TSC2 function [49]. Cully and colleagues reported that p38-activating stresses, such as H_2_O_2_ and anisomycin, were able to activate TORC1 in mammalian and Drosophila tissue culture [50]. This stress-induced TORC1 activation could be blocked by RNAi against mitogen-activated protein kinase kinase 3 and 6 (MKK3/6). p38 was also required for the activation of TORC1 in response to amino acids and growth factors. In another study, Hernández and colleagues reported that reactive oxygen species (ROS) can activate p38 in certain conditions of cell stress, which in turn reduces the level of REDD1 (regulated in development and DNA damage responses 1) [51]. The decline in REDD1 leads to the dissociation of the TSC1/2 via enhanced association of TSC2 with 14-3-3 family members [51]. In addition, González-Terán and colleagues showed that, upon hypertrophy-inducing stimuli, p38γ/δ promote mTORC1 activity and cardiac hypertrophy by phosphorylating DEPTOR and leading its ubiquitination and degradation [52]. Given the well-established crosstalk of p38 with PKA [53,54] and PKG [43,55,56] signaling, it would be interesting to determine whether p38 might also convey the PKA and PKG actions to mTORC1. However, the physiological and pathological context of such regulation are yet to be established.

GSK3β is an established negative regulator of mTORC1 signaling. After the primed phosphorylation of TSC2 by AMPK at S1345, GSK3β phosphorylates TSC2 at several amino acid residues (Thr1329, Ser1333, Ser1337, Ser1341) and results in mTORC1 inhibition [57]. GSK3 activity is inhibited through phosphorylation of serine 21 in GSK3α and serine 9 in GSK3β. These serine residues in GSK3 are within motifs that allow them to be targets of both AKT and PKA [58]. PKA physically associates with, phosphorylates, and inactivates both isoforms of GSK3 [58]. Thus, the activity of GSK3 can be modulated either by growth factors that work through the PI3K-AKT cascade or by hormonal stimulation of G protein-coupled receptors that link to changes in intracellular cAMP levels [58], which will ultimately dictate the mTORC1 activity.

PKA might regulate mTORC2 activity through both direct and indirect mechanisms. As mentioned earlier, a study from our group identified three PKA phosphorylation sites in mTOR, and phosphorylation-resistant mutations of these sites (S1276, S1288, and S2112) resulted in impaired mTORC1 activation as indicated by S6K1 phosphorylation [41]. Thus, it is possible that PKA can regulate mTORC2 activity through the direct phosphorylation of mTOR. In addition, PKA might be able to control mTORC2 activity through inhibiting GSK3. Chen and colleagues reported that GSK-3β phosphorylates the mTORC2 component RICTOR at Serine 1235 in cellular stress conditions [59]. This modification interferes with the binding of AKT to mTORC2 and thus inhibits AKT activity. Koo and colleagues reported that GSK3 is associated with RICTOR and directly phosphorylates the Thr 1695 [60]. This modification results in the recruitment of E3 ubiquitin ligase F-Box and WD repeat domain-containing 7 (FBW7) and leads to RICTOR degradation. In both cases, GSK3 negatively regulates mTORC2 activity. However, in another study, Urbanska and colleagues reported that GSK3 can positively regulate mTORC2 in mature neurons [61], suggesting that there may be important cell-type-specific regulatory pathways.

**Table 1:** Regulation of mTOR by cAMP and cGMP signaling pathways.

No.	Reagents	Signaling Cascade and Effects	Mechanism	Targets	Readouts	Ref.
	**Inhibition by cAMP**					
1	Forskolin, IBMX	Inhibits interleukin-2-dependent mTORC1 activation in IL-2-responsive lymphoid cells	Activates PKA and inhibits PI3K and S6K1 activation	PI3K	p-S6K1	[23]
2	Forskolin, cAMP analogue	Prevents the effect of insulin on mTORC1 activation in 3T3-L1 adipocyte	Inhibits mTOR phosphorylation and activation	mTOR	p-4E-BP	[24]
3	Forskolin, IBMX	Inhibits insulin and amino acid-stimulated mTORC1 activation in MEFs and HEK293 cells	Promotes mTOR complex disassembly and inhibits mTOR catalytic activity	mTOR	p-S6K1, p-4E-BP	[25]
4	Forskolin, IBMX	Inhibits serum-stimulated mTORC2 activation after prolonged cAMP elevation in MEFs and HEK293 cells	Promotes mTOR complex disassembly and inhibits mTOR catalytic activity	mTOR	p-PKB, p-PKC	[25]
5	Forskolin, cAMP analogue	Inhibits mTORC1 activation in Ret/PTC1-positive thyroid carcinoma cell TPC-1	Inhibits the Raf/ERK cascade and depends on PKA	Raf/ERK cascade	p-S6K1, p-4E-BP	[26]
6	cAMP analogue	Inhibits EGF-stimulated mTOR activity in Swiss 3T3, HEK293, COS, and Rat2 cells	Inhibits PI3K activity, PDK1 translocation to plasma membrane, and AKT phosphorylation	PI3K/PDK/AKT cascade	p-S6K1	[27]
7	Glucagon, cAMP analogue	Inhibits insulin- and amino acid-induced mTORC1 activation in serum-free primary rat hepatocytes	NA	NA	p-S6K1, p-4E-BP1	[28]
8	Glucagon	Represses both basal and amino acid-induced mTORC1 activation in perfused rat liver	Activates PKA and enhances phosphorylation of LKB1 Thr172 and AMPK Ser428	LKB1/AMPK pathway	p-S6K1, p-4E-BP1	[29]
9	Glucagon	Acts in a dominant manner to repress insulin-induced mTORC1 signaling in perfused rat liver	Reduces assembly of the mTORC1-eIF3 complex	mTORC1-eIF3 complex	p-S6K1, p-4E-BP1	[30]
10	PTH/PTHrP	Leads to mTORC1 inhibition during skeletal development	Inhibits SIK3 and prevents DEPTOR degradation	DEPTOR	p-S6K, p-S6	[31]
	**Activation by cAMP**					
11	Forskolin, GLP-1RA exenatide	Activates mTORC1 in isolated rat islets	Mobilizes intracellular Ca^2+^ influx and upregulates ATP production	K_ATP_ channel	p-S6K1	[35]
12	Adrenalin, cAMP analogue	Potentiates insulin-stimulated mTORC1 activation in soleus muscles ex vivo	Depends on cAMP and Epac but not PKA	EPAC	p-S6K1	[36]
13	GLP-1, Forskolin	Leads to S6 phosphorylation in the pancreatic beta cell line MIN6 and islets	Depends on PKA phosphorylation of S6 at Ser235/Ser236	S6	p-S6	[37]
14	TSH	Activates mTORC1 in PC Cl3 rat thyroid cells	Acts in part via phosphorylation of PRAS40 Thr246 by PKA and augments 4E-BP1 binding to RAPTOR	PRAS40	p-S6K1, p-4E-BP1	[38]
15	hCG, Forskolin	Activates mTORC1 in theca-interstitial cells	Involved in cAMP-dependent activation of AKT	AKT	p-S6K, p-S6, p-4E-BP1	[39]
16	cAMP	Leads mTORC1 activation in HEK293 cells	Disrupts the interaction of PDE4D with RHEB and increases the interaction between RHEB and mTOR	PDE4D and RHEB	p-S6K1, p-4EBP1	[40]
17	β-AR agonist	Leads to mTORC1 activation in adipocyte and mouse adipose tissue	Direct PKA phosphorylation of RAPTOR Ser791 and mTOR Ser1276, Ser1288, and Ser2112	RAPTOR and mTOR	p-S6K1	[41]
18	GLP-1RA liraglutide	Promotes mTORC1 activation in CHO cells expressing GLP-1R	Direct PKA phosphorylation of RAPTOR Ser791	RAPTOR	p-S6K1	[62]
	**Activation by cGMP**					
19	Natriuretic peptides	Leads to mTORC1 activation in adipocyte and mouse adipose tissue	Direct PKG phosphorylation of RAPTOR Ser791	RAPTOR	p-S6K, p-S6	[42]
	**Inhibition by cGMP**					
20	PKG	Inhibits mTORC1 and promotes autophagy activity	Direct PKG1 phosphorylation of TSC2 Ser1365 and Ser1366, PKG1α C42 oxidation reduces TSC2 phosphorylation and amplifies mTORC1 activity	TSC2	p-S6K1, p-4E-BP1	[44,46]
	**Indirect Regulation via p38**					
21	p38	Activates mTORC1 by inhibiting TSC2	Controls TSC2 Ser1210 phosphorylation through MAPKAPK2 (MK2) and increases 14-3-3 binding	TSC2	p-TSC2	[49]
22	p38β	Leads to TORC1 activation in stressed mammalian and Drosophila tissue culture	Occurs downstream of or in parallel to TSC2 and upstream of Rag activation	ND	p-S6K1, p-4E-BP1	[50]
23	p38	Leads to mTORC1 activation in reperfused heart and reoxygenated or H_2_O_2_-treated cardiomyocytes (NRVMs)	ROS downregulates REDD1 and promotes TSC2/14-3-3 association	TSC2	p-S6	[51]
24	p38γ/δ	Leads to mTORC1 activation in postnatal cardiac hypertrophic growth	Phosphorylates DEPTOR and leads to its degradation and mTOR activation	DEPTOR	p-mTOR, p-S6K1, p-S6	[52]
	**Indirect Regulation via GSK3β**					
25	GSK3β	Phosphorylates TSC2 and results in mTORC1 inhibition	PKA physically associates with, phosphorylates, and inactivates both isoforms of GSK3	TSC2	p-AKT	[57,58]
26	GSK3β	Required for mTORC2 inhibition during ER stress	Phosphorylates RICTOR at Ser1235, interferes with the binding of AKT to mTORC2 and thus inhibits AKT	RICTOR	p-AKT	[59]
27	GSK3β	Required for mTORC2 inhibition	Directly phosphorylates RICTOR at Thr1695, recruits FBW7 and leads to RICTOR degradation	RICTOR	p-AKT, p-SGK1, p-PKCα	[60]
28	GSK3β	Required for mTORC2 activation in response to BDNF or insulin stimulation in cultured neurons and mouse brain	Increases prosurvival signaling	NA	p-S6, p-AKT	[61]

**Abbreviations**: IBMX: 3-Isobutyl-1-methylxanthine; MEFs: mouse embryonic fibroblast; PTH/PTHrP: parathyroid hormone/PTH-related peptide; GLP-1: glucagon-like peptide-1; GLP-1RA: GLP-1 receptor agonist; KATP channels: ATP-sensitive K+ channels; TSH: thyroid-stimulating hormone; hCG: human chorionic gonadotropin; β-AR: β-adrenergic receptor; NRVMs: neonatal rat ventricular myocytes; ER: endoplasmic reticulum; GPCRs: G protein-coupled receptors.

## 3. Role of mTOR Signaling in Cardiometabolic Disease

### 3.1. Role of mTOR Signaling in Obesity

Obesity develops as increased adiposity in fat mass due to a chronic surplus of calorie intake over energy expenditure. According to the guidelines, a body mass index (BMI) over 25 is considered overweight and over 30 is obese [63]. Body weight and composition, and the storage of energy as triglyceride in adipose tissue, are determined by the interaction between genetic, environmental, and psychosocial factors [64,65]. The energy balance is determined by the two arms—energy intake and energy expenditure—and they are controlled by both central and peripheral mechanisms. The central nervous system regulates energy balance by controlling the feed behavior, autonomic nervous activity, and neuroendocrine system [64,65]. The energy expenditure takes the form of physical activity, basal metabolism, and adaptive thermogenesis [64,65]. This part of the review will summarize the studies describing the role of mTOR signaling in obesity and systemic metabolism, with a focus on the adipose tissue and the neuronal control of food intake.

Insulin acts through the PI3K cascade to activate two downstream pathways to mTORC2 as well as via TSC to mTORC1. The dysregulation of mTOR signaling results in insulin resistance, a hallmark of metabolic syndrome [66]. In the fed state, increased blood glucose levels stimulate the pancreas to secrete insulin. Insulin promotes anabolic processes in target tissues to maintain glucose and lipid homeostasis, including lipogenesis, fatty acid import, and glycogen and protein synthesis (reviewed in [1]). During fasting, the drop in nutrients, growth factors, and insulin facilitates the shift of metabolic balance in favor of lipolysis, ketogenesis, and gluconeogenesis (reviewed in [1]). The negative feedback loop between mTORC1 and mTORC2 is tightly regulated under physiological conditions. In obesity, hyperactivation of mTORC1 induced by excessive nutrition and mitogens can lead to phosphorylation of insulin receptor substrate 1 (IRS-1) by S6K1 [67,68,69] and negative regulation of insulin signaling by GRB10 (growth factor receptor bound protein 10) [70,71]. Following phosphorylation at multiple residues, IRS1 is degraded and the insulin signaling is attenuated, leading to insulin resistance and ectopic lipid accumulation in muscle and the liver [67,68,69]. GRB10 inhibits the insulin signaling pathway by disrupting the association of IRS-1/IRS-2 with the insulin receptor [72]. However, GRB10 can also inhibit mTORC1 through a feedback mechanism. mTOR-mediated phosphorylation at Ser501/503 switches the binding preference of GRB10 from the insulin receptor to RAPTOR, leading to the dissociation of RAPTOR from mTOR and the inhibition of mTORC1 signaling [73].

Studies with adipose tissue-specific *Raptor* knockout mice have demonstrated that mTORC1 is essential for adipose tissue function and homeostasis (Table 2). Polak and colleagues reported that adipose-specific *Raptor* knockout mice had substantially less adipose tissue, elevated energy expenditure proposed to be due to mitochondrial uncoupling, were protected against diet-induced obesity, hypercholesterolemia, and insulin resistance [74]. However, this study used the *Fabp4/aP2*-Cre, which shows a leaky expression in other tissues including the brain [75,76,77], and it is difficult to appreciate whether these phenotypes are caused by the adipose tissue, per se, or by defects in the other tissues. Our group reported that mice with mTORC1 impairment through adipocyte-specific deletion of *Raptor* by the *Adipoq*-Cre were refractory to the βAR-dependent induction of uncoupling protein 1 (UCP1) expression and expansion of beige/brite adipocytes in white adipose tissue (WAT) [41]. Mechanistically, PKA directly phosphorylates mTOR and RAPTOR on unique serine residues. Abrogation of the PKA site within RAPTOR disrupted βAR/mTORC1 activation of S6K1 while a phospho-mimetic RAPTOR augmented S6K1 activity [41]. This study suggested that PKA activation of mTORC1 is essential for β-adrenergic stimulation of adipose browning. Lee and colleagues further showed that RAPTOR/mTORC1 loss in adipocytes (*Adipoq*-Cre) causes progressive lipodystrophy and fatty liver disease [78]. Adipose *Raptor* knockout (*Adipoq*-Cre) mice are resistant to high-fat diet (HFD)-induced obesity, but this is due to failed adipose tissue expansion and not increased energy expenditure, which in turn results in severe hepatomegaly associated with hyperphagia and defective dietary lipid absorption [78]. Thus, mTORC1 activity in mature adipocytes is essential for maintaining normal adipose tissue growth and its selective loss in mature adipocytes leads to a progressive lipodystrophy disorder and systemic metabolic disease. In addition, Paolella and colleagues showed that mice with adipocyte *Raptor* deletion (*Adipoq*-Cre) failed to completely suppress lipolysis in the fed state and displayed prominent hypertriglyceridemia and hypercholesterolemia [79]. This study suggested that adipose tissue mTORC1 activity is necessary for appropriate suppression of lipolysis and for the maintenance of systemic lipid homeostasis. In another study, Zhang and colleagues reported that adipose-specific depletion of *Raptor* (*Adipoq*-Cre) increased prostaglandin (PG) production by cyclooxygenase-2 (COX-2) and promoted differentiation of progenitor cells to beige adipocytes [80]. This study identifies adipocyte-derived PGs as key regulators of white adipocyte browning and this was proposed to occur through an mTORC1-dependent mechanism.

mTORC2 signaling also plays an important role in adipose tissue function and homeostasis (Table 2). Early work by Cybulski and colleagues showed that mice with adipose-specific *Rictor* deletion by *Fabp4*/*aP2*-Cre have increased body size due to an increase in the size of non-adipose organs [81]. These mice have a disproportionately enlarged pancreas and are hyperinsulinemic, but they are glucose-tolerant and display elevated levels of insulin-like growth factor 1 (IGF1) and IGF1 binding protein 3 (IGFBP3) [81]. These findings indicate that mTORC2 in adipose tissue plays an unexpectedly central role in controlling whole-body growth. However, as noted earlier, these results must be interpreted with caution due to the use of the *Fabp4/aP2*-Cre driver, which can also alter mTORC2 activity in non-adipose tissues due to this leaky expression [75,76,77]. Tang and colleagues showed that mTORC2 functions in white adipose tissue (WAT) to control expression of the lipogenic transcription factor ChREBPβ (carbohydrate response element-binding protein β). Conditionally deleting *Rictor* in mature adipocytes (Adipoq-Cre) decreases ChREBPβ expression, which reduces DNL in WAT, and impairs hepatic insulin sensitivity [82]. Mechanistically, RICTOR/mTORC2 promotes ChREBPβ expression in part by controlling glucose uptake, but without impairing pan-AKT signaling [82]. Thus, mTORC2 functions in WAT as part of an extra-hepatic nutrient-sensing mechanism to control glucose homeostasis. Hung and colleagues showed that *Rictor* loss in the *Myf5*+ precursor lineage (*Myf5*-Cre) shifts BAT metabolism to a more oxidative and less lipogenic state and protects mice against obesity and metabolic disease [83]. Jung and colleagues showed that conditionally deleting *Rictor* in murine brown adipocytes (*Ucp1*-Cre) inhibits de novo lipid synthesis, promotes lipid catabolism and thermogenesis, and protects against diet-induced obesity and hepatic steatosis [84]. Deleting *Rictor* in brown adipocytes appears to drive lipid catabolism independently of AKT by promoting FoxO1 deacetylation, and this occurs in a pathway distinct from its positive role in anabolic lipid synthesis [84]. These findings suggest a new paradigm of mTORC2 function filling an important gap in our understanding of this less well-understood mTOR complex.

As a crucial arm of the energy balance in obesity, feeding behavior is subjected to short-term and long-term controls for the initiation and termination of meals and the status of energy storage [64,65]. The hypothalamus is a region in the brain that controls appetite and is essential for metabolic homeostasis. The GLP-1 receptor agonists (GLP-1RA) have emerged as the second-generation anti-obesity medications due to their metabolically beneficial effects to stimulate insulin secretion while suppressing food intake [85,86]. Studies by Burmeister and colleagues showed that mTORC1 signaling is also essential in mediating the weight loss effect of GLP-1RA action [87]. Pharmacological GLP-1R activation in the ventromedial hypothalamus (VMH) reduces food intake and body weight, and mTORC1 activity appears necessary for these effects of GLP-1RA [87]. In a recent study, in collaboration with our lab, Le and colleagues showed that GLP-1RA similarly stimulates mTORC1 activity via PKA phosphorylation of RAPTOR at Ser791, and this phosphorylation event is relevant to the weight loss effect of the therapeutic GLP-1RA liraglutide [62]. In addition, knock-in mice expressing Ser791Ala RAPTOR were partially resistant to GLP-1R agonist-induced weight loss. These results support the conclusion that PKA activation downstream of the GLP-1R leads to phosphorylation of RAPTOR at Ser791, which activates mTORC1 signaling in a non-canonical fashion to facilitate the weight-lowering effects of liraglutide. Further studies are needed to investigate whether the RAPTOR S791 phosphorylation is required for the effect of GLP-1RA on insulin secretion.

### 3.2. Role of mTOR Signaling in Fatty Liver Disease

Nonalcoholic fatty liver disease (NAFLD) is a spectrum of liver disease ranging from nonalcoholic fatty liver (NAFL) to nonalcoholic hepatic steatosis (NASH), the latter of which could advance to cirrhosis and hepatocellular carcinoma (HCC) (reviewed in [88]). NAFL is defined by the presence of fat accumulation in >5% of hepatocytes as assessed by histology or non-invasive imaging [89]. NASH is a more severe and progressive form of the disease and is histologically characterized by hepatocyte injury, inflammation, and fibrosis as observed in liver biopsy [90]. The disease progression from NAFL to NASH is involved in multiplex factors, including metabolic dysfunction, genetic variants, age, ethnicity, and diet [91]. In the US, the incidence of NAFLD as of 2017 was estimated at ~25% of the population and the prevalence of NASH was about 3–4% [92], and this number is growing. Of note, NASH is related to a number of different factors and is closely associated with an increased risk of cardiovascular disease, a leading cause of mortality in patients with the disease [93,94]. Despite its prevalence, no medication is currently approved to treat this condition [88].

After feeding, glucose taken up by the liver is converted into acetyl-CoA through a stepwise process of glycolysis, tricarboxylic acid (TCA) cycle and citrate lyase [95]. Hepatic de novo lipogenesis (DNL) is involved in the synthesis of fatty acids (FAs) from acetyl-CoA and their further processing into triacylglycerols (TAG). DNL is controlled by the rate-limiting enzyme acetyl-CoA carboxylase (ACC), which converts acetyl-CoA to malonyl-CoA, and fatty acid synthesis (FASN), which processes acetyl-CoA and malonyl-CoA into palmitate [95]. Palmitate will be further esterified to produce TAG. Of note, malonyl-CoA inhibits carnitine acyltransferase [96], the rate-limiting step in fatty acid oxidation, and it thus diverts metabolites to lipid synthesis. The expression of ACC and FASN are transcriptionally controlled by various transcriptional regulators including sterol response element-binding protein (SREBP), carbohydrate response element-binding protein (ChREBP), and nuclear receptors such as PPARγ, FXR, and LXR [95]. During fasting, hepatic glucose production is increased through the induction of both glycogenolysis and gluconeogenesis. Fasting also stimulates lipolysis in adipose tissue and thus the release of nonesterified FAs (NEFAs). NEFAs are further converted into ketone bodies in the liver via β-oxidation and ketogenesis [97].

mTOR signaling plays an essential role in hepatic lipid metabolism (reviewed in [97]). As the primary hormone that drives hepatic lipogenesis, insulin acts through PI3K-AKT signaling and depends on mTORC1. mTORC1 regulates the expression of a panel of lipogenic genes, including the master regulator SREBPs. The SREBPs belong to the basic helix–loop–helix leucine zipper transcription factor family, and consist of three members, SREBP1a, SREBP1c, and SREBP2. Despite the functional overlap among SREBPs, SREBP1c is primarily responsible for the lipogenic gene expression program in the liver [95,97]. Upon insulin stimulation, SREBPs are translocated from the endoplasmic reticulum (ER) to Golgi, followed by proteomic N-terminus cleavage and shuttle to the nuclei to drive the expression of genes that control cholesterol and FA synthesis [95,97].

mTORC1 controls the activation of SREBPs through both S6K1-dependent and S6K1-independent mechanisms (reviewed in [97]). The S6K1-dependent mechanism of SREBPs’ regulation by mTORC1 is not well understood; however, Lipin1 and CRTC2 (CREB regulated transcription coactivator 2) are two major regulators known to be involved in the S6K1-independent regulation of SREBPs [97]. Lipin1 can shuttle to nuclei in its dephosphorylated form and promote the binding of SREBPs with the nuclear matrix, thus preventing SREBPs from binding and activating the SRE-containing lipogenic genes [98]. Once phosphorylated by mTOR, Lipin1 is restricted in the cytoplasm and is unable to inhibit the SREBPs’ action in the nuclei [98]. CRTC2 can bind to COPII subunit Sec31A and thus disrupt the transport of SREBPs from the ER to Golgi [99]. Upon feeding, CRTC2 is phosphorylated and inactivated by mTOR, thereby attenuating the inhibition effect of CRTC2 on SREBPs’ maturation [99].

Autophagy plays a critical role in shuttling lipid droplets in the hepatocyte to the lysosome for hydrolysis, a process termed lipophagy [100]. mTORC1 controls lipophagy by inhibitory phosphorylation of the machinery of autophagy, including ULK1/2 (UNC-51-like autophagy activating kinase 1/2), ATG13 (autophagy-related 13), ATG14L (autophagy related 14), and the transcriptional regulators of lysosome biogenesis, including TFEB (transcription factor EB) and TFE3 (transcription factor binding to IGHM enhancer 3). Under fed conditions, TFEB is phosphorylated by mTOR and restricted in the cytoplasm by binding to 14-3-3 [101,102,103,104]. During fasting, TFEB is dephosphorylated and translocated to nuclei to promote the expression of autophagic and lysosomal genes [105,106,107]. TFEB also promotes lipid oxidation by upregulating the expression of PPARγ and PGC1α [108]. In addition, TFE3, another member of the MITF/TFE family that is phosphorylated by mTORC1, also regulates autophagy and lysosomal biogenesis gene expression during starvation [109,110].

Given its critical role in lipid metabolism, the role of mTORC1 in NAFLD is well appreciated but turns out to be complicated (Table 2). Deletion of *Raptor* in the liver suppresses DNL through the inhibition of the SREBP-1c-regulated lipogenic gene program and protects mice from hepatic steatosis [98]. However, mTORC1 activation through the hepatocyte deletion of TSC1 also protects the mice from liver steatosis [111,112]. And yet additional studies showed that mice lacking *Raptor* in the liver have increased steatosis [113] and liver injuries [114]. Thus, the role mTORC1 in liver steatosis is still not well understood, which might be due to the complexity of mTORC1 signaling in the context of disease progression [115]. In a recent study, Gosis and colleagues reported that selective inhibition of mTORC1, through the deletion of the RagC/D guanosine triphosphatase-activating protein folliculin (FLCN), protects against NAFLD and NASH [115]. This disease protection is mediated by the activation of TFE3, without affecting the signaling arms mediated by S6K1 and 4E-BP1 [115]. TFE3 inhibits lipogenesis by suppressing proteolytic processing and activation of SREBP-1c and by interacting with SREBP-1c on chromatin [115]. In another study, Uehara and colleagues sought to define the role of hepatic mTORC1 activity in mouse models of NASH and investigate the mTORC1-dependent mechanisms responsible for protection against liver damage in NASH [116]. Utilizing two rodent NASH-promoting diets, they demonstrated that hepatic mTORC1 activity was reduced in mice with NASH, whereas under conditions of insulin resistance and benign fatty liver, mTORC1 activity was elevated [116]. Mice with acute liver-specific loss of TSC1 have an improved profile of NASH and are protected against hepatic inflammation and fibrosis by constitutive mTORC1 activity. In another study, Kim and colleagues reported a mechanism where mTORC1-independent (‘free’) RAPTOR negatively regulates hepatic AKT activity and lipogenesis through the β-TrCP-mediated degradation of the AKT phosphatase, PHLPP2 [117]. Forced PHLPP2 expression ameliorates hepatic steatosis in diet-induced obese mice. These data suggest that the balance of free and mTORC1-associated RAPTOR governs hepatic lipid accumulation and uncovers the potentially therapeutic role of PHLPP2 activators in NAFLD. Taken together, these studies indicate that the role of mTORC1 in the pathophysiology of NAFLD and NASH needs to be interpreted with caution.

Studies also demonstrated that mTORC2 plays important roles in liver lipid homeostasis (Table 2). Hagiwara and colleagues reported that fed mice with liver-specific *Rictor* deletion displayed a loss in AKT Ser473 phosphorylation and reduced glucokinase and SREBP1c activity in the liver, leading to constitutive gluconeogenesis and impaired glycolysis and lipogenesis [118]. These liver-specific defects resulted in systemic hyperglycemia, hyperinsulinemia, and hypolipidemia. Expression of constitutively active AKT2 in mTORC2-deficient hepatocytes restored both glucose flux and lipogenesis, whereas glucokinase overexpression rescued glucose flux but not lipogenesis. Thus, mTORC2 regulates hepatic glucose and lipid metabolism via insulin-induced AKT signaling to control whole-body metabolic homeostasis. Yuan and colleagues reported that mice lacking *Rictor* in the liver (LrictorKO) failed to inhibit hepatic glucose output in response to insulin [119]. LrictorKO mice also resisted the development of hepatic steatosis on a high-fat diet and manifested lower levels of serum cholesterol, expression of SREBP-1c and SREBP-2, and genes of fatty acid and cholesterol biosynthesis. LrictorKO mice had defects in insulin-stimulated AKT Ser-473 and Thr-308 phosphorylation, leading to decreased phosphorylation of AKT substrates. LrictorKO mice also manifested defects in insulin-activated mTORC1 activity, evidenced by decreased S6 kinase and Lipin1 phosphorylation.

Dysregulation of mTOR signaling in the liver also results in cancer progression. Guri and colleagues reported that mice lacking *Tsc1* and *Pten* specifically in the liver (termed L-dKO mice) exhibited concomitant activation of mTORC1 and mTORC2 signaling and displayed hepatosteatosis progressing to hepatocellular carcinoma (HCC) [120]. Longitudinal proteomic, lipidomics, and metabolomic analyses revealed that hepatic mTORC2 promotes de novo fatty acid and lipid synthesis, leading to steatosis and tumor development. In particular, mTORC2 stimulated sphingolipid (glucosylceramide) and glycerophospholipid (cardiolipin) synthesis. Inhibition of fatty acid or sphingolipid synthesis prevented tumor development, indicating a causal effect in tumorigenesis. Increased levels of cardiolipin were associated with tubular mitochondria and enhanced oxidative phosphorylation. Furthermore, increased lipogenesis correlated with elevated mTORC2 activity and HCC in human patients. Thus, mTORC2 promotes cancer via the formation of lipids essential for growth and energy production.

### 3.3. Role of mTOR Signaling in Cardiac Remodeling

The mTOR pathway regulates both physiological and pathological processes in the heart (reviewed in [121,122]). It is needed for embryonic cardiac development and for postnatal cardiac remodeling in conditions of pressure overload. Under unstressed conditions, mice with constitutive cardiac deletion of mTOR die in utero or during the perinatal period [123]. Mice with cardiac-specific deletion of mTOR in the adult stage showed a reduced life span, along with fatal dilated cardiomyopathy, molecular features of mitochondrial dysfunction, and increased apoptosis and autophagy [123].

mTORC1 is a critical regulator of cardiac hypertrophy and remodeling (Table 2). Cardiac hypertrophy is induced by mechanical stress, such as pressure or volume overload, and by neurohormonal factors, such as angiotensin II (Ang-II) and adrenergic stimulation [121]. mTOR signaling is activated by the stimuli that trigger cardiac hypertrophy and promote both the adaptive and maladaptive cardiac growth [121]. In cardiomyocytes, rapamycin inhibits the Ang-II-induced upregulation of S6K1 and suppresses the increases in S6K1 activation and protein synthesis in response to isoproterenol or phenylephrine treatment [124]. Thus, the G protein-coupled receptor (GPCR) signaling has a major impact on mTORC1 activation during hypertrophy development [121]. Mice with inducible cardiac deletion of RAPTOR in adults develop marked cardiac dysfunction in response to pressure overload induced by transverse aortic constriction (TAC) without the development of compensatory hypertrophy [125,126]. Constitutive mTORC1 activation promotes the transition from adaptive to maladaptive hypertrophy [121]. In mouse models of pressure or volume overload, pharmacological mTORC1 inhibition is protective and improves cardiac remodeling [127,128,129]. RAPTOR haploinsufficiency attenuates heart failure induced by pressure overload or Gαq overexpression [130]. This protective effect is mediated by 4E-BP1-independent mechanisms, such as the effect of mTORC1 on mitochondria and metabolism [130]. The heart undergoes structural and functional changes during aging [121]. Rapamycin administration has been reported to increase lifespan, improve cardiac function, and results in a reduced gene expression signature involving inflammation, hypertrophy, and contractile function [131,132].

The activity of mTORC1 can be modified by other pathways during cardiac stress conditions. Studies showed that p38 MAPK contributes to the maladaptive hypertrophy during stress conditions by enhancing mTORC1 activity. As described earlier, reactive oxygen species (ROS) can activate p38, which in turn reduces the level of REDD1 [51]. The decline in REDD1 leads to the dissociation of the TSC1/2 via the enhanced association of TSC2 with 14-3-3 family members [51]. In addition, p38γ/δ MAPKs can phosphorylate DEPTOR and promote its ubiquitination and degradation [52]. p38γ/δ MAPK knockout mice show reduced mTOC1 activity and reduced postnatal heart growth; however, they are protected from angiotensin II-induced hypertrophy [52]. Recent studies revealed that PKG signaling can negatively regulate mTORC1 signaling in pathological cardiac remodeling. As described earlier, PKG activation reduces cardiac hypertrophy in mice who underwent pressure overload [44]. Mechanistically, PKG activates TSC2 by direct phosphorylation at Ser1365 and 1366 and results in mTORC1 inhibition and enhanced autophagy [44]. Gain- or loss-of-function mutations at either of the two adjacent serine residues in TSC2 can bidirectionally control mTORC1 activity stimulated by growth factors or hemodynamic stress and consequently modulate cell growth and autophagy [44]. Homozygous knock-in mice that express a phosphorylation-silencing mutation in TSC2 develop worse heart disease and have higher mortality after sustained pressure overload of the heart, owing to mTORC1 hyperactivity that cannot be rescued by PKG1 stimulation. In addition, PKG activity is reduced in response to hypertrophic stimuli due to the oxidation at cystine 42 [45]. The PKG1α cysteine-42 redox state controls mTORC1 activation in pathological cardiac hypertrophy [46]. Mice with a redox-dead cysteine 42 to serine mutation show improved cardiac hypertrophy and dysfunction in response to pressure overload due to the TSC2 activation and mTORC1 inhibition [46]. Furthermore, soluble guanylyl cyclase-1 (sGC-1) activation and phosphodiesterase (PDE) 9 inhibition but not PDE5 inhibition can offset the growth-hormone-stimulated mTORC1 activity regardless of PKG-1α redox state and phosphorylation of TSC2 S1365 [46].

Myocardial ischemia or energy stress leads to mTORC1 inhibition and protective adaption such as autophagy, which in turn limits myocardial infarction [121]. RHEB is inhibited in response to ischemia or glucose deprivation, leading to mTORC1 inhibition [133]. Forced expression of RHEB induces cell death and ER stress, inhibits autophagy, and increases infarct size. In addition, GSK3β inhibition also leads to mTORC1 activation and thus restricts autophagy, resulting in increased myocardial ischemic injury [134]. Restoration of autophagy rescued the deleterious effect of RHEB upregulation or GSK3β inhibition. Of note, mTORC1 shows differential roles during ischemia and reperfusion [121]. mTORC1 inhibition by rapamycin before ischemia reduces the ischemia/reperfusion (I/R) injury while it does not confer any protection in the reperfusion phase [134,135]. In fact, endogenous mTORC1 is activated during the reperfusion phase and limits I/R injury [134,136]. mTORC1 activation is detrimental during chronic ischemia. In rat models undergoing myocardial infarction (MI), mTORC1 inhibition by everolimus reduced adverse remodeling and infarct size and promoted autophagy [129]. The mTORC1 substrate S6K1 is activated in the heart during MI in mice and the pharmacological inhibition of S6K1 attenuated myocardial remodeling [137].

Unlike the adverse effect of mTORC1, studies have shown that mTORC2 activation promotes compensatory cardiac growth and limits maladaptive hypertrophy (Table 2). Constitutive deletion of *Rictor* in cardiomyocytes induces cardiac dysfunction and dilation in response to pressure overload [138]. *Rictor* deletion in adults also leads to cardiac dysfunction in mice undergoing TAC [138]. In addition, activation of mTORC2 protects against age-induced cardiac abnormalities. Systemic heterozygous deletion of *Rictor* decreases lifespan in male mice, suggesting a protective role of mTORC2 against aging [139]. Furthermore, mTORC2 activation also has a protective role in chronic ischemia. In mice undergoing MI, loss of RICTOR increases myocardial damage and remodeling [140]. Of note, PRAS40 inhibition improves cardiac function and post-infarction remodeling through the mTORC2-induced AKT activation [140]. Thus, induction of a shift from mTORC1 to mTORC2 activation is protective for heart ischemic disease [121].

**Table 2:** Metabolic and cardiac phenotypes of *Raptor* and *Rictor* knockout mice.

No.	Genes	Tissues	Models	Phenotypes	Ref.
	**Adipose tissue**				
1	*Raptor*	Adipocyte *Fabp4/aP2*-Cre	HFD	Substantially less adipose tissue, protected against diet-induced obesity, hypercholesterolemia, and insulin resistance	[74]
2	*Raptor*	Adipocyte *Adipoq*-Cre	HFD	Progressive lipodystrophy with hepatic steatosis and insulin intolerance, resistant to diet-induced obesity, severe hepatomegaly	[75]
3	*Raptor*	Adipocyte *Adipoq*-Cre	HFD	Fails to completely suppress lipolysis in the fed state and displays prominent hypertriglyceridemia and hypercholesterolemia	[79]
4	*Raptor*	Adipocyte *Adipoq*-Cre	Cold, βAR agonist	Refractory to the β-adrenergic stimulation of UCP1 expression and expansion of beige/brite adipocytes in WAT	[41]
5	*Raptor*	Adipocyte *Adipoq*-Cre	Cold, HFD	Increased prostaglandin (PG) production by COX-2 and promotes differentiation of progenitor cells to beige adipocytes	[80]
6	*Raptor*	Global S791A knock-in	GLP-1R agonist	Partially resistant to GLP-1R agonist liraglutide-induced weight loss, lesser reduction in fat mass, lower energy expenditure	[62]
7	*Rictor*	Adipocyte *Fabp4/aP2*-Cre	HFD	Increased body (non-adipose organ) size, hyperinsulinemia but glucose-tolerant, elevated levels of IGF1	[81]
8	*Rictor*	Adipocyte *Adipoq*-Cre	HFD	Normal body growth, insulin intolerance, impaired adipose insulin signaling results in reduced glucose uptake and de novo lipogenesis	[82]
9	*Rictor*	Brown adipocyte *Myf5*-Cre	HFD	Decreased fat mass and adipocyte size, decreased lipogenesis but elevated mitochondrial activity in brown fat, protected against obesity and metabolic disease	[83]
10	*Rictor*	Brown adipocyte *Ucp1*-Cre	Cold, βAR agonist, HFD	Increased cold tolerance, inhibits de novo lipid synthesis, promotes lipid catabolism and thermogenesis, and protects against diet-induced obesity	[84]
	**Liver**				
11	*Raptor*	Hepatocyte *Albumin*-Cre	Western diet	Gained less weight, reduced fasted liver size, suppresses hepatic de novo lipogenesis, protects mice from hepatic steatosis and hypercholesterolemia	[98]
12	*Raptor*	Hepatocyte AAV-TBG-Cre	Fasting and refeeding	Smaller liver with increased steatosis, higher hepatic TAG and decreased TAG secretion under fasting conditions	[113]
13	*Raptor*	Hepatocyte *Albumin*-Cre	HFD, hepatic carcinogen	Increased liver injuries, aberrant regeneration, enhanced fibrosis, inflammation, and hepatocarcinogenesis	[114]
14	*Rictor*	Hepatocyte *Albumin*-Cre	HFD	Smaller liver with lower glycogen and TG content, hepatic insulin resistance, dysregulated hepatic gluconeogenesis, glycolysis and de novo lipogenesis	[118]
15	*Rictor*	Hepatocyte *Albumin*-Cre	HFD	Failed to inhibit hepatic glucose output, glucose intolerance and insulin resistance, resistant to hepatic steatosis on a high-fat diet	[119]
	**Heart**				
16	*Raptor*	Cardiomyocyte *MHC*-Cre	TAC	Acute cardiac dysfunction in response to overload, metabolic switch from fatty acid to glucose oxidation, abnormal mitochondria, increased apoptosis, and autophagy	[126]
17	*Raptor*	Cardiomyocyte *MHC*-Cre heterozygote	TAC	Attenuates heart failure induced by pressure overload or Gαq overexpression	[130]
18	*Rictor*	Cardiomyocyte *MHC*-Cre	TAC	Accelerates cardiac dysfunction after aortic constriction, reduces PKC protein levels, does not affect hypertrophy, fibrosis, or metabolic gene expression	[138]
19	*Rictor*	Cardiomyocyte AAV9 shRNA	MI	Increases myocardial damage and remodeling	[140]

**Abbreviations**: TBG: thyroxine binding globulin; TAC: transverse aortic constriction; MHC: α-myosin heavy chain; MI: myocardial infarction.

## 4. Conclusions

In this review, we summarize the recent findings demonstrating the regulation of the mTOR signaling network by cAMP and cGMP, as well as by their associated cyclic nucleotide-dependent protein kinases PKA and PKG. Although these signaling crosstalks could be mediated by various mechanisms as reported in previous studies, further effort is needed to elucidate the role of these signaling events in physiological and disease conditions.

## Data Availability

No new data were created.

## Abbreviations

mTOR	mechanistic target of rapamycin;
RAPTOR	regulatory-associated protein of mTOR;
RICTOR	rapamycin-insensitive companion of mTOR;
DEPTOR	DEP domain-containing mTOR-interacting protein;
mLST8	mammalian lethal with SEC13 protein 8;
PRAS40	proline-rich AKT substrate of 40 kDa;
mSIN1	mammalian stress-activated protein kinase-interaction protein 1;
PROTOR	protein observed with RICTOR;
TSC1/2	tuberous sclerosis complex 1/2;
RHEB	Ras homolog mTORC1 binding;
RAGs	Ras-related GTPases;
IRS	insulin receptor substrate;
PI3K	phosphoinositide 3-kinase;
PIP3	phosphatidylinositol 3,4,5-triphosphate;
PTEN	phosphatase and tensin homolog;
PDK1	phosphoinositide-dependent protein kinase 1;
GRB10	growth factor receptor bound protein 10;
AKT;	protein kinase B;
ERK	extracellular signal-regulated kinase;
RSK	ribosomal S6 kinase 1;
TNF	tumor necrosis factor;
IKKβ	inhibitor of nuclear factor kappa-B kinase subunit beta;
AMPK	AMP-activated protein kinase;
WNT	wingless and lnt-1;
GSKβ	glycogen synthase kinase 3β;
REDD1	regulated in development and DNA damage responses 1;
p38 MAPK	p38 mitogen-activated protein kinases;
MK2	MAPK activated protein kinase 2;
ROS	reactive oxygen species;
NO	nitric oxide;
ANP	atrial natriuretic peptide;
BNP	B-type natriuretic peptide;
GPCR	G protein-coupled receptor;
GC	guanylyl cyclase;
sGC	soluble guanylyl cyclase;
cAMP	cyclic adenosine monophosphate;
cGMP	cyclic guanosine monophosphate;
PKA	cAMP-dependent protein kinase;
PKG	cGMP-dependent protein kinase;
SIK3	salt-inducible kinase 3;
FKBP12	FK506-binding protein of 12 kDa;
PIP2	phosphatidylinositol 4,5-bisphosphate
PKA	protein kinase A;
PKG	protein kinase G;
GPCRs	G protein-coupled receptors;
EPAC	exchange protein directly activated by cAMP (EPAC);
LKB1	liver kinase B1;
PDE	phosphodiesterase;
NPs	natriuretic peptides;
TBG	thyroxine binding globulin;
TAC	transverse aortic constriction;
MHC	α-myosin heavy chain;
MI	myocardial infarction;
IBMX	3-Isobutyl-1-methylxanthine;
MEFs	Mouse embryonic fibroblast;
PTH/PTHrP	parathyroid hor-mone/PTH-related peptide;
GLP-1	Glucagon-like peptide-1;
GLP-1RA	GLP-1 receptor agonist;
KATP channels	ATP-sensitive K+ channels;
TSH	thyroid-stimulating hormone;
hCG	human chorionic gonadotropin;
β-AR	β-adrenergic receptor;
NRVMs	neonatal rat ventricular myocytes;
ER	endoplasmic reticulum;
GPCRs	G protein-coupled receptors.
DNL	de novo lipogenesis;
NAFLD	nonalcoholic fatty liver disease;
NAFL	nonalcoholic fatty liver;
NASH	nonalcoholic hepatic steatosis;
HCC	hepatocellular carcinoma;
UCP1	uncoupling protein 1;
WAT	white adipose tissue.
